# Targeted Radionuclide Detection of Malignant Tumors Using Affibody

**DOI:** 10.32607/actanaturae.27677

**Published:** 2025

**Authors:** O. D. Bragina, A. A. Nesynov, E. Yu. Sitnikova, S. V. Patalyak, S. M. Deyev

**Affiliations:** Tomsk National Research Medical Center of the Russian Academy of Sciences, Tomsk, 634009 Russia; National Research Tomsk Polytechnic University, Tomsk, 634050 Russia; Siberian State Medical University, Tomsk, 634050 Russia; Shemyakin-Ovchinnikov Institute of Biorganic Chemistry of the Russian Academy of Sciences, Moscow, 117997 Russia

**Keywords:** Malignant tumors, theranostics, targeted radionuclide diagnostics, alternative scaffold proteins, affibodies

## Abstract

This review examines the potential applications of affibody molecules in various fields of biotechnology and clinical medicine. Consideration is given to the high affinity and specificity of affibody molecules for selected molecular targets, as well as their potential for the in vivo visualization of various malignant tumors. Significant attention is paid to preclinical and clinical studies of affibody conjugates with various radioisotopes for targeted radionuclide tumor imaging, which is particularly relevant in addressing challenges encountered during the diagnosis and treatment of these patients. Clinical trials demonstrate that radiopharmaceuticals are well-tolerated and effective for the assessment of tumor process prevalence and the determination of HER2/neu status in breast cancer patients, supporting further research.

## INTRODUCTION

The International Agency for Research on Cancer (IARC) indicates that approximately 20 million new cancer cases are diagnosed annually around the globe, with 9.7 million deaths reported in 2022. A trend of annual increases at these rates has been observed. Population forecasts suggest that by 2050, the incidence of malignant tumors (MT) will reach 35 million cases [1]. In the Russian Federation, 674,000 instances of MT were documented in 2023, representing an 8% increase over the figures for 2022 [2]. Given the significant prevalence and socioeconomic impact of cancer, research into the development of more targeted MT diagnostic methods, novel drugs, and strategies to overcome antitumor therapy resistance is essential [3, 4, 5].

The critical step in oncology diagnostics involves acquiring tumor tissue for subsequent histological confirmation. This stage significantly influences sub sequent therapeutic strategies, prognosis, and the necessity for supplementary investigations, includ ing a molecular genetic analysis [6]. Tumor samples may be acquired through core biopsy and excisional biopsy, with the latter entailing complete tumor re moval via diagnostic surgery [7, 8]. The methodolo gies employed are uniformly invasive and potentially distressing, with some instances necessitating patient recovery and rehabilitation, this entailing supple mentary financial expenditures. Hence, thoracoscopic and laparoscopic manipulations require patient hos pitalization and anesthesia, and they do not preclude the potential for procedural failure stemming from the tumor’s anatomical site, the presence of numer ous metastatic foci, and the patient’s unwillingness to undergo the manipulation [9, 10]. The issue of whether immunohistochemical analysis of additional tumor structures is necessary, considering potential receptor status differences from the primary tumor and the limitations of routine application, requires further investigation.

Furthermore, several difficulties are inherent in the histological and immunohistochemistry (IHC) analyzes of tumor tissues, stemming from the complexities of determining tumor cell origins in poorly differenti ated and anaplastic lesions [13], as well as the subjec tive nature of parameter assessment [14]. Analysis of statistical data reveals that the rate of disagreement in diagnoses among pathologists could be as high as 30%, which can be attributed to diagnostic challenges, human factors, and the use of additional staining pro cedures [15, 16].

The limitations of conventional radiological and morphological techniques, the necessity of invasive interventions, a substantial economic burden, and po tential challenges in results interpretation and repro ducibility necessitate the development and adoption of supplementary diagnostic approaches. This ad justment aims to broaden diagnostic and therapeutic possibilities for individuals afflicted with malignant neoplasms, thereby fostering enhanced longevity and improved quality of life. The evolution of new meth odologies utilizing small molecules, specifically those exhibiting antibody-like characteristics and tumor an tigen tropism, is considered to be of significant im portance.

## MALIGNANCY THERANOSTICS

With the developments in fundamental oncology, it has become crucial to determine the molecular genet ic parameters of the tumor. This enables new points of application of drug therapy to be identified, bring ing us closer to the concept of personalized medi cine, i.e., to the determination of treatment based on the biological features of the tumor of each patient with maximum efficiency [17]. Theranostics, a rapid ly evolving field in personalized medicine, integrates diagnostic procedures, such as identifying tumor cell molecular targets and therapy indications, with tar geted therapeutic interventions based on previous ly detected tumor growth markers in the patient. Adoption of the theranostics approach on a larger scale has the potential to improve therapy response rates, lower the incidence of adverse events, increase both overall and relapse-free survival, enhance pa tient quality of life, and lessen the economic burden on healthcare provision [18].

In 1998, John Funkhouser, then the CEO of PharmaNetics, used the term “theranostics” for the f irst time to refer to the business strategy of his com pany, which centered on the creation of diagnostic panels for the subsequent prescription of targeted pharmaceutical treatments [19]. But the concept of employing a single molecule for both cancer diagno sis and treatment had been established some decades earlier. A significant advancement in the development of theranostics was the discovery and widespread ap plication of radioactive iodine in the treatment of thy roid cancer [20]. The pretreatment imaging of the tu mor with the iodine-123 isotope aided in improved treatment strategy planning for radioiodotherapy. Thus, the combination of iodine-123 and iodine-131 was the first radioisotope pair used in theranostics. Progress in this area has been significantly impact ed by advances in radiochemistry and enhanced in strumental investigation techniques [21]. It is impor tant to acknowledge that the journal Theranostics (www.thno.org) has been providing summaries of per tinent data in this domain for over ten years. This f ield has experienced a recent surge in development, attributable to the use of radionuclides.

Classification includes photo-, sono-, chemo-, nano- and radiotherapy, contingent on diagnostic methods and therapeutic agents [22]. Targeted radionuclide di agnostics has potential as the only area of theranos tics implemented in clinical practice. This approach is based on radioisotope-labeled “targeting” molecules that selectively attach themselves to receptors present on the surface of tumor cells. The recording of isotope radiation using specialized equipment can facilitate both the anatomical and molecular evaluation of tu mor presence in patients with malignant neoplasms, eliminating the need for further invasive procedures, and enabling repeated examinations at the stages of primary diagnosis and treatment [23, 24, 25].

Radiopharmaceuticals (RPHs) incorporate diverse molecular structures as targeting modules, such as full-size monoclonal antibodies, antibody fragments, and synthetic framework molecules/alternative scaf fold proteins (ASPs), with the latter exhibiting signifi cant potential in radionuclide diagnostics [26, 27, 28, 29].

**Alternative scaffold proteins**

The accelerated evolution of technologies for the clon al selection of polypeptides via binding from substan tial libraries has enabled the development of a new class of binding proteins, employing protein engineer ing approaches. In order to reduce immunogenicity, these structures were designed using various scaf folds which differ in both size and structural organ ization from the original immunoglobulin. A range of non-immunoglobulin affinity proteins have been documented, based on several surface-located amino acid residues present in secondary structure elements or unstructured loops, subsequently selected through different display platforms [30].

The structure of these compounds typically com prises a constant scaffold part (constant region) and a variable region. The first component contains a pair of α-helices or β-sheets forming a rigid tertiary struc ture and maintains the conformational stability in herent in protein scaffolds. The second component comprises several open loops or residues within rigid secondary structures that permit specific binding to different target molecules through structural ligand receptor pairing or chemical interactions [31].

The biodistribution and tumor penetration capa bilities of ASP are primarily determined by their compact size (4–15 kDa). This results in a significant reduction in the time interval between the adminis tration of the radiopharmaceutical medicinal product (RPhMP) and the start of imaging study, improves the degree of drug accumulation in the tumor, and influ ences the choice of a radioisotope suitable for specific research goals and timing. ASPs are also typically known for their robust ability to withstand environ mental conditions. The absence of disulfide bridges in the ASP structure, typical for antibodies, as well as a sufficiently dense structure, determines its high ther mal stability, stability in acidic and alkaline conditions, and resistance to proteolysis. Furthermore, additional structures can be introduced into protein scaffolds through chemical synthesis for conjugation with phar maceuticals or diagnostic agents. The solubility and robust physicochemical stability of small protein scaf folds are typically advantageous for in vivo applica tion. Given the aforementioned characteristics, scaf fold proteins can be regarded as versatile compounds for modification and production [32].

Currently, representatives of the alternative scaf fold protein class include affibody molecules, affil ins, anticalins, avimers, DARPins, Kunitz-type in hibitor domains, and albumin-binding domains (e.g., ADAPTs), among others. Each of these is at various stages of investigation, with an ongoing search for po tential points of future clinical application.

## AFFIBODY IN BIOTECHNOLOGY AND CLINICAL MEDICINE

Affibody, a synthetic molecule, is an example of an alternative protein framework with a domain struc ture. The affibody molecule has a Z-domain at its core, which is the peptide domain of protein A of Staphylococcus aureus [33]. The spatial structure of the Z-domain, which comprises 58 amino acid residues and has a relatively low molecular mass (~6.5 kDa), is formed by three alpha-helices that create a bundle. Affibody molecules exhibit good structural stability, resistance to proteolysis, high temperatures (around 90°C), and acidic and alkaline conditions (pH from 2.5 to 11) [34]. Affibody combinatorial libraries can be generated by randomly modifying genes that encode 13 amino acid residues in the first and second helices of the Z-domain. For this purpose, phage, cellular, ri bosomal and mRNA displays are used. Libraries are combined with the target antigen to facilitate the se lection of molecules, which are subsequently washed to eliminate unbound ligands, leaving only those pep tides bound to the ligand. The primary targeting mol ecules obtained can be subjected to further re-ran domization to increase their affinity for a particular target [35]. Variants of interest can be produced in bacterial, yeast, and cell systems. Moreover, due to their small size, affibodies can also be created via pep tide synthesis methods [36]. Introducing a functional izing group at the N- or C-terminus of a peptide ena bles the acquisition of a molecule with the properties required for a specific application, such as radioiso tope labeling for radionuclide diagnostics or a cyto toxic group for targeted treatment [37, 38].

The unique properties of affibody molecules make them of considerable interest in diagnostic and clinical medicine, as well as diverse biomedical applications.

**Fig. 1:**
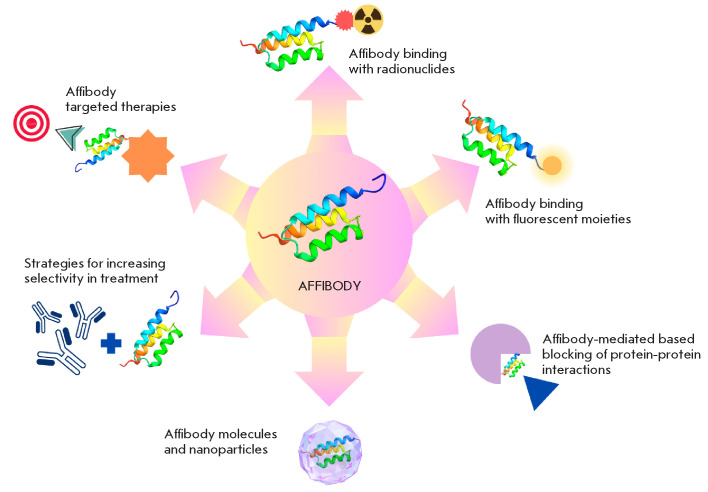
Biotechnological and clinical applications of affibody molecules

Consequently, the possibility of employing affibody molecules conjugated with fluorescent fragments for the bioluminescent detection of malignant lesions is actively being explored. This method offers several key advantages: high sensitivity, the absence of toxicity and the avoidance of invasive procedures, as well as cost-effectiveness [39].

Affibody molecules can also function as a barrier between interacting proteins, offering a novel approach to treating diseases caused by these interactions. Viral diseases can serve as an example. Viral spike proteins are important targets for vaccine and antiviral drug development. For instance, an affibody molecule has been shown to have high specificity and affinity for the RBMFP protein (a product synthesized from the SARS-CoV-2 protein), and the interaction of the affibody molecule with the Receptor Binding Motif (RBM) leads to the neutralization of the SARS-CoV-2 pseudovirus infection [40].

Conjugates of affibody molecules with various types of nanoparticles are considered promising agents for the therapy and imaging of malignant neoplasms [41]. For example, an affibody conjugate with the contrast agent nanobullbe is used for binding HER2 and IR783 and developing a method for ultrasound detection of HER2-positive breast cancer [42]. The potential therapeutic effect of high-affinity probes such as Gd@C-dots-Cys-ZEGFR:1907 against EGFR in the therapy of non-small cell lung cancer and PFH/AGM-CBA/HSV-TK/liposome (PAHL)- affibody in the therapy of HER2-positive breast cancer is being studied [43, 44]

Of particular importance is the development of affibody-based targeting drugs. A Phase III clinical trial of Izokibep®, an IL-17 inhibitor, was initiated in 2022 to examine its effects on numerous complement-dependent diseases such as psoriatic arthritis, uveitis, ankylosing spondylitis, and hidradenitis suppurativa [45, 46].

**Affibody for targeted radionuclide diagnostics of malignancies**

The studies predominantly explore the potential of using affibody as a basis for radiopharmaceuticals for the targeted imaging of malignant neoplasms of various localizations. The selection of the imaging method is crucial for the radionuclide diagnosis of malignancies. This is due to the unique characteristics of each radioisotope, among which are the half-life (T_1/2_), the type of radiation (positron or gamma radiation), and the method of production (generator or cyclotron) [47, 48]. Currently, single-photon emission computed tomography (SPECT) and positron emission tomography (PET) are used for radiation detection, with the choice depending on the isotope incorporated into the radiopharmaceutical preparation (RPH) (Table 1). Presently, the radionuclides most frequently used in conjunction with affibody molecules encompass ^68^Ga (T_1/2_ = 68 min), ^99m^Tc (T_1/2_ = 6.02 h), ^18^F (T1/2 = 109.8 min), among others. The longlived radionuclides used are ^66^Ga (T1/2 = 9.9 h), ^64^Cu (T1/2 = 12.7 h), ^188^Re (T1/2 = 1 7 h ), ^89^Zr (T_1/2_ = 78.4 h), ^111^In (T_1/2_ = 2.81 days ), ^177^Lu (T_1/2_ = 6.7 days), ^125^I (T_1/2_ = 60 days), ^57^Co (T_1/2_ = 271.8 days) [49].

**Table 1:** Radioisotopes for radionuclide diagnostics using PET or SPECT

Radioisotope T_1/2_	Emission type	Production	method
^68^Ga	68 min	β+, γ	Generator
^99m^Tc	6.02 h	γ	Generator
^18^F	109.8 min	β+	Cyclotron
^66^Ga	9.9 h	β+, γ	Cyclotron
^64^Cu	12.7 h	β+, β–, γ	Cyclotron
^188^Re	17 h	β–, γ	Generator
^89^Zr	78.4 h	β+	Cyclotron
^111^In	2.81 days	γ	Cyclotron
^177^Lu	6.7 days	β–, γ	Cyclotron
^125^I	60 days	γ	Cyclotron
^57^Co	271.8 days	γ	Cyclotron

Radioconjugates are synthesized to target receptors that are overexpressed on the surface of tumor cells in many malignant pathologies. These receptors are not only involved in the pathogenesis of malignant tumors but also represent additional therapeutic options for cancer patients (Table 2). For instance, preclinical studies are currently underway on radioconjugates targeting the ligand of the programmed cell death receptor PD-L1. This receptor is a transmembrane protein that regulates the cellular immune response, and the expression of PD-L1 by tumor cells or cells of the tumor microenvironment leads to the inhibition of the cellular immune response. This allows tumor cells to evade apoptosis associated with the cytotoxic action of T-lymphocytes. PD-L1 expression has been detected in various tumors, including melanoma, lung cancer, breast cancer, bladder cancer, pancreatic cancer, and ovarian cancer [50, 51, 52]

**Table 2:** Affibody-based radiopharmaceuticals in various stages of clinical and preclinical trials

RPH	Imaging method	Target receptor	Research phase	Authors, year
[^99m^Tc]Tc-PDA-Affibody	SPECT	PD-L1	Preclinical	Liang et al., 2022 [53]
[^99m^Tc]Tc-AC12-GGGC	SPECT	B7-H3	Preclinical	Oroujeni et al., 2022 [56]
[^99m^Tc]Tc-SYNT-179	SPECT	B7-H3	Preclinical	Oroujeni et al., 2023 [57]
[^68^Ga]Ga-DOTA-ZTRI	PET	PDGFRβ	Preclinical	Cai et al., 2023 [58]
[^18^F]AlF-NOTA-HER2	PET	HER2	Preclinical	Han et al., 2022 [64]
[^99m^Tc]Tc-(HE)3ZHER2:V2	SPECT	HER2	Preclinical	Hu et al., 2024 [68]
[^111^In]In-ABY-002	SPECT	HER2	Clinical (Phase I)	Baum et al., 2010 [72]
[^68^Ga]Ga-ABY-002	PET	HER2	Clinical (Phase I)	Baum et al., 2010 [72]
[^111^In]In-ABY-025	SPECT	HER2	Clinical (Phase I)	Sörensen et al., 2014 [73]
[^68^Ga]Ga-ABY-025	PET	HER2	Clinical (Phase I)	Sörensen et al., 2016 [74]
[^68^Ga]Ga-NOTA-Mal-Cys-MZHer342	PET	HER2	Clinical (Phase I)	Miao et al., 2022 [75]
[^68^Ga]Ga-ABY-025	PET	HER2	Clinical (Phase II)	Alhuseinalkhudhur et al., 2023 [76]
[^68^Ga]Ga-ABY-025	PET	HER2	Clinical (Phase II)	Altena et al., 2024 [77]
[^99m^Tc]Tc-ZHER2:41071	SPECT	HER2	Clinical (Phase I)	Bragina et al., 2023 [78]

For example, Liang et al. [53] assessed the pharmacokinetics of [^99m^Tc]Tc-PDA-affibody, its toxicity profile, and the potential for in vivo imaging of PD-L1-positive tumors via SPECT at 30, 60, and 120 min post-injection. The tumor was observed to exhibit a fairly rapid accumulation of RPH after 30 min. Nevertheless, an imaging interval of 1–2 h was considered optimal, given the overall drug distribution. The disadvantages associated with this pharmaceutical agent include poor SPECT resolution, substantial drug concentration in the kidneys, thyroid, and gastrointestinal tract, attributed to the binding of unconjugated technetium-99m oxide.

Another promising target for targeted imaging is the B7-H3 (CD276) receptor, a transmembrane protein from the immune checkpoint molecule family that has a co-activating or co-inhibitory effect on T-lymphocytes. In normal tissues, this protein is expressed at a rather low level, but overexpression of this protein has been observed in some tumors [54]. These include prostate cancer, renal cell and urothelial cancer, ovarian cancer, and others. Within tumor tissue, this protein exerts a pro-oncogenic effect by suppressing the antitumor immune response. Given the active development of immunotherapy, this receptor is considered a viable target, thereby making the development of specific diagnostics for B7-H3 overexpression in tumor tissue a priority [55].

Oroujeni et al. [56] investigated the drug [ ^99m^Tc]Tc-AC12-GGGC in ovarian and breast cancer cell lines. A Ramos lymphoma cell line lacking B7-H3 expression served as a negative control. The B7-H3-positive xenograft cells demonstrated a sixfold increase in drug accumulation compared to the control group. However, a minimal absolute amount of drug accumulation was observed in the tumor. SPECT imaging, conducted four hours after RPH injection, demonstrated visualization of the xenografted B7-H3-positive tumor, while in the negative control group the tumor was not visualized. High accumulation of [99mTc]Tc-AC12-GGGC was also noted in tissues such as the kidneys and liver.

Oroujeni et al. [57] also investigated the possibility of improving the detection of B7-H3 overexpression with the radiopharmaceutical preparation [ ^99m^Tc]Tc-AC12-GGGC by increasing the affinity of the affibody molecule. After they were generated via phage display, three daughter molecules were labeled with technetium-99m and evaluated in mouse models, along with the original AC12 molecule. As a result, the SYNT-179 molecule was selected, as it possesses superior characteristics: higher tumor accumulation, lower accumulation in normal tissues with an improved tumor-to-organ ratio, and lower RPH accumulation in the liver. The study demonstrated that affinity maturation improved molecular biodistribution and imaging performance, and that the optimized Affibody protein exhibited enhanced performance in targeting B7-H3 overexpression.

In a preclinical study, Cai et al. [58] investigated the use of a trimeric affibody molecule labeled with ^68^Ga([^68^Ga]Ga-DOTA-Z_TRI_) for PET diagnostics of hepatocellular carcinoma (HCC). [^68^Ga]Ga-DOTA-Z_TRI_ exhibits high affinity for platelet-derived growth factor receptor type beta (PDGFRβ), which is expressed on the surface of pericytes, cells found within the walls of small blood vessels. In normal blood vessels, pericytes are covered by an intact endothelium. However, in tumors, the architecture of the vascular walls is disrupted, resulting in areas of pericytes not covered by endothelium, which renders PDGFRβ on their surface accessible for detection [59]. Therefore, it was hypothesized that PDGFRβ could serve as a potential biomarker for HCC, which is a highly vascularized neoplasm, suggesting that this receptor could be overexpressed in HCC compared to normal liver tissue.

In the initial stage, PDGFRβ was validated as a biomarker for HCC and the trimeric affibody ZTRI was found to have high affinity for PDGFRβ. In addition, the PET data indicated that the accumulation of [^68^Ga]Ga-DOTA-ZTRI correlated directly with PDGFRβ expression by tumor cells; therefore, the drug actively accumulated in PDGFRβ-positive HCC cells in laboratory animals. At the same time, no accumulation of [^68^Ga]Ga-DOTA-ZTRI was detected in healthy liver tissues. Thus, the high potential of the radiopharmaceutical preparation [^68^Ga]Ga-DOTA-ZTRI for PET diagnostics of HCC and the rationale for its further study and implementation in clinical practice were demonstrated.

**Affibody for the detection of HER2-positive malignancies**

Targeted therapy for malignant diseases often focuses on human epidermal growth factor receptor type 2 (HER2/neu), a tyrosine kinase receptor that is key to cell differentiation, proliferation, and apoptosis. HER2 overexpression, primarily attributed to ERBB2 gene amplification, has been identified in breast, gastric, pancreatic, lung, endometrial, ovarian, bladder, colorectal cancer, and various other tumor localizations [60].

Current approaches for determining HER2 status include IHC and fluorescence in situ hybridization (FISH) techniques. As per the 2023 ASCO/CAP guidelines, a HER2/neu expression result is deemed negative in the absence of staining or with faint, sporadic membrane staining (0 and 1+) and is considered positive with intense, complete circumferential membrane staining in more than 10% of tumor cells (3+). For ambiguous cases (2+), the result is confirmed via amplification of the HER2 gene using FISH and an ERBB2(17q12)/SE17 DNA probe (Kreatech, USA) [61].

The scientific community is currently directing its attention toward investigating targeted radionuclide detection using affibody to evaluate HER2/neu receptor expression in gastric and ovarian cancers. This is due to the unique anatomical challenges and the pursuit of supplementary therapeutic strategies within these oncological contexts. Gastric cancer is often diagnosed at late stages when surgical treatment is not feasible, requiring the molecular biological parameters of the tumor to be determined for selecting a systemic therapy option. HER2 expression is detected in 17–20% of gastric cancer cases. However, a very high level of heterogeneity in HER2 expression is observed (14–79% by IHC and 23–54% by IHC + FISH). Furthermore, the HER2 status of a tumor can change during anti-HER2 therapy, causing difficulties in assessing the effectiveness of the ongoing treatment, as performing multiple biopsies is associated with risks of complications and is not always an option [62, 63].

Han et al. [64] researched the possibility of targeted detection using the [^18^F]AlF-NOTA-HER2 preparation. The HER2-positive cell line was NCI-N87, whereas the HER2-negative cell line was MKN74. In vitro studies demonstrated the accumulation of the RPH in question in HER2-expressing cells. In vivo, [ ^18^F]AlF-NOTA-HER2 was found to rapidly accumulate in HER2-positive xenografts and to be quickly eliminated from the blood, primarily by the kidneys. Within normal tissues, the highest accumulation was observed in bones and kidneys, which was a significant drawback of this molecule, as the high level of absorbed radioactivity requires nephroprotection. The comparison of [^18^F]AlF-NOTA-HER2 and 68Ga-NOTA-HER2 demonstrated a benefit in using fluorine-18 over gallium-68, due to its longer half-life (109.8 min vs. 67.7 min, respectively), which provided more time for the study. Additionally, the shorter positron diffusion range of fluorine-18 results in improved resolution in PET imaging.

Ovarian cancer presents multiple diagnostic and therapeutic challenges attributable to its high recurrence and distant metastasis rates, in addition to the large proportion of cases diagnosed in advanced stages [65, 66]. Until recently, anti-HER2 therapy was not used to treat tumors of this type, owing to adverse outcomes with trastuzumab. Nonetheless, research in this area has been resumed due to the development of monoclonal antibody conjugates with cytostatics. For example, the DESTINY-PanTumor02 study investigating the efficacy of trastuzumab-deruxtecan therapy in various solid tumors reported objective response rates (ORR) in 63-64% of patients with HER2-positive ovarian cancer, which makes it promising in determining the HER2 status in this oncopathology [67].

Hu et al. conducted a feasibility study on affibody molecules in ovarian cancer utilizing [ ^99m^Tc]Tc-(HE)_3_ Z_HER2:V2_ [68]. The results demonstrated elevated compound accumulation in HER2/neu-overexpressing tumors, whereas tumors lacking HER2/neu expression did not exhibit drug accumulation. A disadvantage of [^99m^Tc]Tc-(HE)_3_ Z_HER2:V2_ discovered during the study was its high accumulation in the kidneys, which could potentially lead to nephrotoxicity. However, it is assumed that this shortcoming may be remedied through enhanced patient hydration in clinical settings.

**Affibody for diagnosing HER2-positive breast cancer**

Overexpression of the HER2 receptor occurs in 15–20% of breast cancer (BC) cases and has traditionally been associated with a more aggressive course and, consequently, a worse prognosis. Nevertheless, the use of targeted anti-HER2 therapy has improved the overall survival of patients with HER2-positive cancer, approaching the prognosis observed in more favorable molecular genetic subtypes [69]. Currently employed in clinical practice are drugs including trastuzumab, pertuzumab, and lapatinib, in addition to a new class: conjugates of monoclonal antibodies and cytostatics (trastuzumab-emtansine and trastuzumab-deruxtecan) [70, 71].

To date, multiple trials have been performed using affibody as a targeting agent for radionuclide diagnosis of HER2/neu status in individuals with operable, locally advanced and metastatic breast cancer.

In 2005, Baum et al. [72] performed the first clinical study of the indium-111- and gallium-68-labeled affibody molecule ABY-002 to evaluate safety, pharmacokinetics, and the feasibility of imaging tumor foci in breast cancer patients. Following administration, [ ^111^In]In-ABY-002 and [^68^Ga]Ga-ABY-002 demonstrated swift clearance from the circulation, enabling SPECT and PET imaging to commence within 2–3 h postinjection. It was also shown that these drugs were effective in radionuclide tumor imaging: all patients demonstrated an accumulation of the investigated compounds in HER2-positive tumors. Additionally, in one case, [^68^Ga]Ga-ABY-002 allowed muscle metastasis (quadriceps) to be detected, which was not identified by 18F-FDG PET. Notwithstanding the favorable outcomes, [^111^In]In-ABY-002 and [^68^Ga]Ga-ABY-002 present drawbacks, including elevated accumulation in the liver and kidneys, thereby substantially impeding tissue visualization within those areas. For example, it proved impossible to detect liver metastasis in one patient and an adrenal gland metastasis in another.

The next stage involved studying a second-generation affibody molecule (ABY-025). The study by Sörensen et al. [73] included seven patients with metastatic breast cancer: five with the HER2-positive and two with the HER2-negative disease. As in the study with ABY-002, the administration of [^111^In]In-ABY-025 was safe and not associated with adverse events. According to SPECT data, in addition to clear visualization of HER2-positive tumors, weak accumulation of the studied drug was observed in HER2-negative foci, attributable to the presence of a certain amount of the HER2 receptor on the surface of tumor cells. During the study, metastatic foci in the liver, not detected using the ABY-002 molecule, were visualized. An interesting finding of this analysis was the detection of a brain tumor metastasis, previously undetected by ^18^F-FDG-PET, as well as the identification of a HER2-negative tumor metastasis in a patient with a positive HER2 status of the primary breast tumor. The primary limitation observed was the failure to identify metastatic nodes below 1 cm

Since the low visualization of small tumor foci could be due to the low resolution of SPECT, a study of the [^68^Ga]Ga-ABY-025 drug using PET was conducted. An analysis of 16 patients with metastatic BC, conducted by Sörensen et al. [74], demonstrated good visualization of small-sized foci, enabling the detection of breast cancer metastases in the liver, bones, lymph nodes, brain, and other organs. Furthermore, in two patients after the [^68^Ga]Ga-ABY-025 study, the HER2 status in the primary breast tumor was changed from negative to positive. In most cases, differences in HER2 expression between the primary tumor and metastatic foci were found.

Miao et al. [75] investigated the usage of t h e g alli um - 6 8 -l a b el e d a f fi b o d y m ol e c ul e NOTA-Mal-Cys-MZHer342 in a clinical trial that included 24 BC patients. An important component of the analysis included the utilization of PET/CT scans “on demand” by oncologists to address complex diagnostic issues. This approach was used in six patients to differentiate between metastases and two concurrent breast cancers, or concurrent MT of a different origin. In all cases, the SUVmax of [^68^Ga]Ga-NOTA-Mal-Cys-MZHer342 [ ^68^Ga]Ga-NOTA-Mal-Cys-MZHer342 in tumor foci was compared with the results of immunohistochemical examination. According to the analysis, [ ^68^Ga]Ga-NOTA-Mal-Cys-MZHer342 usage allowed researchers to detect HER2 overexpression in tumor tissue with a 91.7% specificity and to detect negative HER2 expression with an 84.6% specificity, with a conversion of HER2/neu status from positive to negative observed in seven patients.

A Phase II study conducted by Alhuseinalkhudhur et al. [76] included 19 patients with primary stage II–III BC scheduled for neoadjuvant therapy with dual targeted anti-HER2 blockade, and 21 patients with metastatic breast cancer undergoing systemic therapy. The premise for this analysis was the assumption that the accumulation of [ ^68^Ga]Ga-ABY-025 could be a predictor of an early tumor response to ongoing anti-HER2 therapy. According to the study design, patients underwent ^18^F-FDG-PET/CT before and after two courses of chemo/targeted therapy to assess early metabolic response, and [68Ga]Ga-ABY-025 before treatment.

A repeat biopsy was performed in all cases on a tumor focus to evaluate the HER2 status relative to the treatment. In a cohort of 12 patients, a comparison of PET imaging with [^68^Ga]Ga-ABY-025 and biopsy data uncovered a discrepancy in HER2/neu status, which was ascribed to several factors. These encompassed challenges in tumor material acquisition, such as the observed prevalence of negative HER2 expression in positive PET scans when samples originated from the liver or bone, intratumoral heterogeneity, and drug binding impediments to HER2 receptors, which garnered specific attention due to the association between a positive biopsy result with diminished [^68^Ga]Ga-ABY-025 accumulation and a poorer prognosis within the metastatic breast cancer cohort. Furthermore, the study revealed an inverse relationship between the number of prior treatment lines and the metabolic response to the current therapy: increased prior treatment lines corresponded to a higher RPH accumulation threshold for a metabolic response. However, given that this correlation was observed in only 30% of cases and that no significant concordance was found between PET with Affibody and biopsy results, a phase III study with [^68^Ga]Ga-ABY-025 was deemed unwarranted.

Altena et al. [77] performed the initial clinical trial involving [^68^Ga]Ga-ABY-025, which explored the potential to visualize metastatic breast cancer with HER2-low tumors. The study included eight patients with negative (IHC 1+) and an equivocal (IHC 2+, FISH negative) HER2 status, as well as two patients with no HER2 expression (IHC 0), previously determined based on primary breast tumor biopsy results. In one patient, the absence of HER2 expression was accompanied by minimal accumulation of [ ^68^Ga]Ga-ABY-025, which correlated with the IHC results. In another case, the drug accumulation was higher and did not correspond to the status determined by HER2 biopsy. A detailed study of the tumor focus revealed heterogeneity of HER2 expression with higher RPH accumulation at the periphery and low accumulation in the center. Therefore, it is probable that the biopsy sample originated from the central, “cold” region, whereas the primary tumor exhibited a HER2-low status. In two other cases, high accumulation was observed in previously unverified foci, which could indicate HER2 overexpression and the potential for prescribing first-line anti-HER2 therapy. In the remaining eight cases, proportional accumulation of [^68^Ga]Ga-ABY-025 according to HER2 status was noted, consistent with the results of numerous studies of this RPH. Thus, PET using [^68^Ga]Ga-ABY-025 can serve as an additional diagnostic tool for selecting patients eligible for therapy with antibody-drug conjugates.

**Fig. 2:**
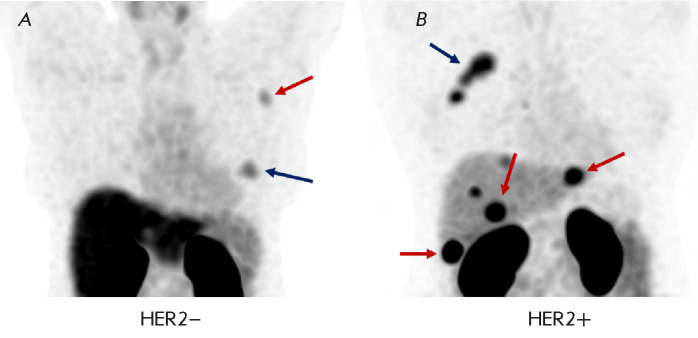
Accumulation of [^99m^Tc]Tc-ZHER2:41071 in breast cancer patients 2 h after its administration at a dose of 1,000 μg: (A) – patient with HER2-negative breast cancer (blue arrow indicates breast tumor; red – metastatic axillary lymph node); (B) – patient with HER2-positive breast cancer (blue arrow indicates breast tumor; red – liver metastases)

In the Russian Federation, Bragina et al. were the first to implement a clinical trial utilizing the radiopharmaceutical [^99^mTc]Tc-ZHER2:41071 [78] for the targeted radionuclide diagnostics of HER2-positive breast cancer, using affibody molecules. The research involved 31 BC patients without prior local or systemic therapies. In all patients, the safety, tolerability, and pharmacokinetics of the drug were assessed, with the accumulation of the RPH by the tumor compared with the results of IHC/FISH. The patients were divided into three cohorts depending on the administered dose of [^99^mTc]Tc-ZHER2:41071: 500, 1,000 and 1,500 µg. All patients showed good tolerability of RPH at all stages of dynamic follow-up. Additionally, at a dose of 1,000 μg, the drug exhibited enhanced pharmacokinetic properties two hours after administration, along with superior breast tumor separation rates contingent upon HER2/neu status. The [^99^mTc]Tc-ZHER2:41071 preparation exhibited a low level of accumulation in normal liver tissue, thereby enabling the visualization of a liver metastasis in one patient, which was later verified through contrast-enhanced computed tomography. A clinical example of the use of RPH is shown in Fig. 2.

## CONCLUSION

Worldwide, the incidence and mortality rates of malignant tumors are notably high. In this context, the diagnostic stage, involving the investigation of clinical- instrumental, morphologic, and molecular parameters, is of particular importance in identifying the optimal strategies for local and systemic treatment [6]. Given the challenges involved in one-step assessment of tumor process prevalence, the necessity of numerous invasive interventions, the financial implications, and the possibility of subjective interpretation, of significant relevance is the introduction of supplementary patient examination methods for those with malignant conditions. Targeted radionuclide imaging shows great potential, because it enables both anatomical staging and the analysis of tumor nodule molecular characteristics, ultimately leading to improved examinations and fewer invasive procedures [23, 24].

This review highlights the potential for affibodies to be used as beneficial agents in biotechnology and clinical medicine, especially in the context of bioluminescent ultrasound imaging and in developing antiviral and targeted therapies [38, 39, 40]. At present, most studies focus on radio conjugates of affibody with varied isotopes for targeted imaging of malignant tumors in various locations. Preclinical trials have consistently demonstrated a strong affinity of Affibody for molecular targets like epidermal growth factor receptor type 2 (HER2/neu) [59], programmed cell death receptor ligand (PD-L1) [52], B7-H3 receptor (CD276) [58], platelet-derived growth factor receptor type beta (PDGFR), and other receptors, as well as suitability for tumor imaging using PET and SPECT techniques.

The findings from several inpatient clinical trials of affibody for HER2-positive breast cancer are compelling [71, 72, 73, 74, 75], demonstrating excellent radiopharmaceutical tolerability and no adverse effects throughout dynamic monitoring. The clinical significance of visualizing breast tumor structures, regional lymph nodes, and distant organs and tissues, alongside HER2/neu expression, is notable, with results comparable to immunohistochemical and FISH analyses [76, 77]. The analysis performed clearly demonstrates the high potential of using alternative non-immunoglobulin framework proteins, like affibody molecules, in clinical applications.

## Abbreviations

MT: malignant tumor
IHC: immunohistochemistry
RPH: radiopharmaceutical
ASP: alternative scaffold proteins
RPD: radiopharmaceutical drug
SPECT: single-photon emission computed tomography
PET: positron emission tomography
HCC: hepatocellular carcinoma
FDG: fluorodeoxyglucose
FISH: fluorescence in situ hybridization
